# Antifungal Effects of *Citrus maxima* Cultivar Tubtim-Siam Peel Extract Against *Malassezia pachydermatis* Isolated from Dogs

**DOI:** 10.3390/pathogens15050479

**Published:** 2026-04-29

**Authors:** Watcharapong Mitsuwan, Juthatip Jeenkeawpieam, Ratchadaporn Boripun, Noppharat Tanthanathipchai, Ozioma Forstinus Nwabor, Phirabhat Saengsawang

**Affiliations:** 1 Akkhraratchakumari Veterinary College, Walailak University, Nakhon Si Thammarat 80160, Thailand; watcharapong.mi@wu.ac.th (W.M.); juthatip.je@wu.ac.th (J.J.); ratchadaporn.bo@wu.ac.th (R.B.); noppharat.ta@wu.ac.th (N.T.); 2 One Health Research Center, Walailak University, Nakhon Si Thammarat 80160, Thailand; 3 Center of Excellence in Innovation of Essential Oil and Bioactive Compounds, Walailak University, Nakhon Si Thammarat 80160, Thailand; 4 Department of Public Health, Maxwell School of Citizenship and Public Affairs, Syracuse University, Syracuse, NY 13244, USA; ofnwabor@syr.edu; 5 Department of Microbiology and Immunology, Faculty of Veterinary Medicine, Kasetsart University, Bangkok 10900, Thailand

**Keywords:** *Citrus maxima*, essential oil, ethanolic extract, *Malassezia pachydermatis*, prototypic topical solution, Tubtim-Siam pomelo

## Abstract

Otitis externa in dogs is primarily caused by *Malassezia pachydermatis*. Treatment involves antifungal and antiseptic agents; however, resistance among causative organisms has been noted. Pomelo (*Citrus maxima*) is a source of bioactive compounds with antimicrobial activity. Its extract mainly includes essential oils, which are mostly applied for alternative treatment for *M. pachydermatis*. The study aimed to investigate the anti-*M. pachydermatis* effects of pomelo peel extracts and their potential use in topical solutions for canine infections. *M. pachydermatis* was isolated from dogs and confirmed with Matrix-Assisted Laser Desorption/Ionization Time-of-Flight Mass Spectrometry (MALDI-TOF/MS). Antifungal susceptibility of *M. pachydermatis* to itraconazole was evaluated. Phytochemicals of essential oil and crude extract from *C. maxima* peel were determined using Gas Chromatograph–Mass Spectrometry (GC-MS/MS). In addition, the antifungal activity of the extracts was assessed using an agar plate dilution assay. The essential oil was formulated into a prototypic topical solution, and its effects on *M. pachydermatis* were observed in vitro. The prevalence of *M. pachydermatis* was 42%, with 53% having yeast on both ear sides. The minimum inhibitory concentrations (MIC) of itraconazole, essential oil, and crude extract to *M. pachydermatis* were 0.03–0.25 µg/mL, 1.0% *v*/*v*, and >200 mg/mL, respectively. The prominent phytochemicals in peel extracts were meranzin hydrate and D-limonene, identified in the crude extract and essential oil, respectively. Moreover, a topical solution containing essential oils inhibited *M. pachydermatis* growth and showed destructive effects on the yeast cell wall at higher concentrations. The essential oil exhibits antifungal activity against *M. pachydermatis*, primarily due to the high concentration of D-limonene. The growth was inhibited completely at MIC, observed over a 5-day period. Furthermore, the prototypic topical solution demonstrated an anti-*M. pachydermatis* effect. These findings suggest potential veterinary applications for pomelo peel extract, though further studies are necessary to assess stability, mechanism of action, and industrial suitability.

## 1. Introduction

Otitis externa in dogs and cats is an ear disease mainly caused by *Malassezia* yeasts [1]. *Malassezia* yeasts can cause dermatitis inside the ear canal and ear pinna areas. Common clinical signs of dogs with otitis externa typically present as erythema of the external ear canal, often accompanied by seborrheic ear discharge [2]. Otitis externa is more commonly associated with *Malassezia* than other yeasts and bacteria [3]. Treatment strategies for *Malassezia* dermatitis include both topical and systemic therapies, primarily involving antiseptic and antifungal applications [4,5]. In addition, treatment failure with antifungal drugs used for *Malassezia* dermatitis therapies, such as clotrimazole, miconazole, ketoconazole, and itraconazole, has been reported [6,7,8]. Antifungal resistance in *Malassezia* yeasts has also been reported in vitro [9,10].

*Malassezia pachydermatis* is an opportunistic yeast that primarily infects the skin of animals, especially dogs and cats [4]. Otitis externa and dermatitis are the main skin infections caused by *M. pachydermatis* globally [11]. Azole antifungal drugs, such as itraconazole, are typically used to treat *M. pachydermatis* infections [6]. The mechanism of the azole group is ergosterol biosynthesis inhibition via the impairment of sterol-14α-demethylase [12]. In addition, *M. pachydermatis* has increasingly been reported as an azole-resistant yeast [6,7]. An alternative treatment is a way to improve antifungal resistance in *M. pachydermatis* strains. Medicinal plants and their extracts represent alternative treatments that play an increasingly important role in the medical and veterinary fields [13,14]. Remarkably, the use of medicinal plants and extracts helps reduce antifungal drug use and the development of antifungal drug resistance.

Pomelos (*Citrus maxima*) are globally cultivated and valued for their nutritional and medicinal benefits, attributed to their abundant phytochemical composition [15]. *C. maxima* cultivar Tubtim-Siam is a pomelo specifically grown in southern Thailand, particularly in Pak Phanang district, Nakhon Si Thammarat province. This cultivar variation of pomelo has ruby-colored pulp, and this part is used for consumption. Nevertheless, its peel is typically discarded as waste. Most compounds in pomelo peels present health-related effects, including antimicrobial activity, anti-inflammation, anti-cancer, and antioxidant properties [16,17,18]. Main antimicrobial compounds in pomelo peels include monoterpenes, sesquiterpenes, alcohols, aldehydes, esters, oxides, and nootkatone [18]. However, the application of pomelo peel essential oil extracts has primarily been focused on humans, and with limited application in veterinary medicine. The objectives of this study were to determine the anti-*M. pachydermatis* activity of *C. maxima* cultivar Tubtim-Siam peel extracts and to evaluate essential oil-containing prototypic topical solutions against *M. pachydermatis* isolated from dogs.

## 2. Materials and Methods

### 2.1. Ethical Approval

Animal handling and laboratory protocols in this study were approved by the Walailak University Institutional Biosafety Committee (WU-IBC) and the Walailak University Institutional Animal Care and Use Committee (WU-IACUC) under the approval IDs of WU-IBC-67-062 and WU-ACUC-67079, respectively.

### 2.2. Isolation of M. pachydermatis from the Ear Canal of Dogs

A sterile cotton swab was used for sample collection from the ear canal of 50 dogs. The sample size was calculated using a previously described formula [19]. Briefly, a previous *Malassezia* species prevalence in dogs [20], 80% sensitivity, 80% specificity, 25% desired precision, and 95% confidence interval were used for calculation settings. The swab was inoculated on HiCrome™ *Malassezia* agar (HiMedia^®^, HiMedia Laboratories, Mumbai, India) and then incubated at 37 °C for 48–72 h. Colonies exhibiting mauve to purple were selected to subculture on Sabouraud dextrose agar (HiMedia^®^, HiMedia Laboratories, Mumbai, India) supplemented with 0.05% chloramphenicol (Bio Basic, Bio Basic Inc., Markham, ON, Canada) and 0.05% cycloheximide (Bio Basic, Bio Basic Inc., Markham, ON, Canada). Colonies on Sabouraud dextrose agar supplemented with 0.05% chloramphenicol and 0.05% cycloheximide were screened for yeast cell morphology using methylene blue staining and then observed under a light microscope. In addition, MALDI-TOF MS was performed following a previously described method [21]. Suspected *M. pachydermatis* colonies were further identified using a Matrix-Assisted Laser Desorption/Ionization Time-of-Flight Mass Spectrometry (MALDI-TOF MS) (MALDI Biotyper^®^, Bruker Daltonik GmbH, Bremen, Germany) at the Office of Scientific Instruments and Testing, Prince of Songkla University, Thailand. Briefly, a 3-day-old *M. pachydermatis* suspected single colony was cultured on Sabouraud dextrose agar. The colony was recultivated overnight, and the culture was then used for protein extraction following Bruker’s standard protocol for protein extraction. The MALDI-TOF MS spectral profile of each sample was compared to the spectra library (MSP). The samples that revealed a score value ≥ 2.00 were identified as the corresponding microorganism, as previously described in bacterial and yeast species-level identification studies [22,23,24]. Confirmed *M. pachydermatis* colonies were kept in Sabouraud dextrose broth supplemented with 30% glycerol and stored at −80 °C until used.

### 2.3. Antifungal Susceptibility Test of Itraconazole

A total of 43 confirmed *M. pachydermatis* isolates were cultivated on Sabouraud dextrose agar and subsequently subcultured in Müller-Hinton broth (HiMedia^®^, HiMedia Laboratories, Mumbai, India) supplemented with 2% glucose (Loba Chemie™, Loba Chemie Ltd., Mumbai, India). The inoculum was adjusted to 2.4 McFarland (3 × 10^6^ CFU/mL) using a densitometer (DEN-1, SIA Biosan, Riga, Latvia). The adjusted inoculum was then diluted into a concentration of 3 × 10^3^ CFU/mL. The diluted inoculum was tested with itraconazole ranging from 0.01 to 16 µg/mL using broth microdilution in a 96-well plate. The tested medium and 1% DMSO were used as a growth control, while yeast-free medium served as a negative control. The plate was incubated at 32 °C for 48 h. Then, 0.03% resazurin was added to each well (Glentham^®^ Life Sciences, Corsham, UK) and incubated at 32 °C for 24 h. The minimum inhibitory concentration (MIC) was defined as the lowest itraconazole concentration that completely inhibited the growth of *M. pachydermatis*. In addition, a total of 5 µL of inoculum of each well was dropped on Sabouraud dextrose agar and incubated at 32 °C for 72 h. The lowest concentration presenting no growth on Sabouraud dextrose agar was defined as the minimum lethal concentration (MLC). Moreover, the determination of MIC and MLC was performed in triplication.

### 2.4. Extraction and Phytochemical Determination of Essential Oil and Crude Extract from C. maxima Cultivar Tubtim-Siam Peel

**Figure 1:**
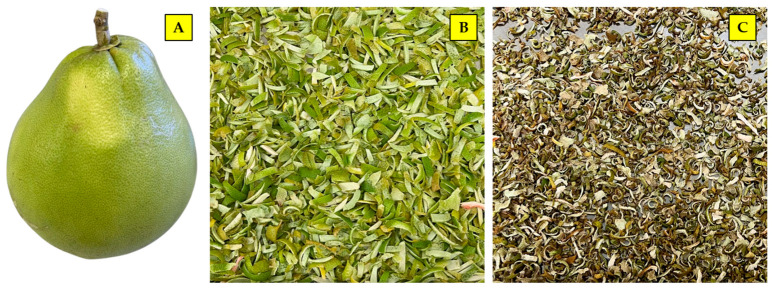
Whole fruit (**A**), fresh flavedo (**B**), and dried flavedo (**C**) of *C. maxima* cultivar Tubtim-Siam.

Whole mature pomelos were pretreated with tap water, 2% sodium bicarbonate, and distilled water to remove debris and agricultural chemicals from their peel. Fresh green peels (flavedo) were used for essential oil extraction and ethanolic extraction. The dried peel ethanolic extraction procedure was performed following a previous study [22]. Whole *C. maxima* cultivar Tubtim-Siam fruit is presented in Figure 1A. For ethanolic extraction, flavedo was dried at 40 °C and ground into powder. The powder was soaked in 95% ethanol for 7 days. The extracted solution was filtered and evaporated using a rotary evaporator at 40 °C under 140 mbar. In addition, the extraction for essential oil was performed following a previously described method with modifications [25]. Briefly, a total of 2000 g of the flavedo was separated and subjected to volatile oil extraction using an essential oil determination apparatus at 60 °C for 12 h. The condensed essential oil was collected and stored at −20 °C until further use. Fresh pomelo peel and dried pomelo peel are presented in Figure 1B and Figure 1C, respectively. The phytochemical composition of both the crude extract and essential oil was determined using Gas Chromatography–Mass Spectrometry/Mass Spectrometry (GC-MS/MS) at the Office of Scientific Instruments and Testing, Prince of Songkla University, Thailand.

### 2.5. Determination of Minimum Inhibitory Concentration and Minimum Lethal Concentration of Ethanolic Extract and Essential Oil of Pomelo Peel

MIC and MLC values of the ethanol extract and essential oil from pomelo peel were determined by an agar dilution assay. The agar dilution method was selected for this study because it is better suited for hydrophobic compounds, particularly essential oils. The initial attempt using a modified broth microdilution assay failed due to poor dispersion. Despite the addition of Tween 80 as an emulsifier, the essential oil separated and led to unreliable results. The agar dilution method, adapted from established protocols, allows more uniform distribution and improves contact between the microorganism and the compound [26,27,28]. The agar dilution protocol for essential oil was performed following previous studies with some modifications [27,28]. A total of 15 *M. pachydermatis* isolates with an itraconazole MIC of 0.25 µg/mL (the maximum MIC and MLC values of tested itraconazole for 43 *M. pachydermatis* isolated from dogs) from individual dogs were included for testing with both ethanolic extract and essential oil of pomelo peel. Briefly, the tested concentration of crude extract ranged from 3.125 to 200 mg/mL and was prepared in molten Sabouraud dextrose agar supplemented with 1% Tween 80. In addition, the essential oil was prepared in the same medium with a range of final concentrations of 0–4% *v*/*v* essential oil. The solidified medium with different concentrations of extract and essential oil was tested with *M. pachydermatis* using the drop plate technique. The tested inoculum was adjusted to a final concentration of 3 × 10^6^ CFU/mL (2.4 McFarland), and a total of 5 µL of inoculum (1.5 × 10^4^ CFU/drop) was dropped onto each concentration of crude extract and essential oil. The plates were then incubated at 32 °C for 5 days, and the presence of yeast colonies was observed daily. Moreover, the experiments were performed in triplicate.

### 2.6. Effect of Pomelo Peel Essential Oil Containing Prototypic Topical Solution on M. pachydermatis

The essential oil was used to prepare a prototypic topical solution. Different essential oil concentrations were prepared for each group. The control group contained the base components of the prototypic topical solutions, including 202.5 µL of glycerol, 202.5 µL of propylene glycol, 25 µL of ethanol (with a final ethanol concentration of 5% *v*/*v* across all treatments), 45 µL of phosphate-buffered saline (pH 7.4; with the PBS volume adjusted depending on the treatment), and 25 µL of essential oil at the specified concentration for each treatment (25% of stock essential oil for treatment 1, 50% of stock essential oil for treatment 2, and 100% of stock essential oil for treatment 3). Moreover, treatment groups included treatment 1 (final essential oil concentration: 12.5%), treatment 2 (final essential oil concentration: 25%), and treatment 3 (final essential oil concentration: 50%). Each treatment was tested on a 2-day-old *M. pachydermatis* colony on Sabouraud dextrose agar by dropping 5 µL of the solution daily directly onto the colony for 3 days. The direct testing of yeast colonies was performed in triplicate. The treated colony was picked up and placed on a sterile 1 × 1 cm^2^ glass slide containing sterile phosphate-buffered saline (pH 7.4). After air drying, the slide was fixed in 2.5% glutaraldehyde for 1 h. The fixed slide was dehydrated in a series of ethanol (20–100%). The dehydrated sample was dried using a critical point dryer (CPD; K850 Critical Point Dryer, Quorum Technologies, Ashford, Kent, UK) and then coated with gold using a gold sputter coater (Cressington Sputter Coater 108 Auto, Cressington Scientific Instruments Inc., Watford, UK). The prepared sample was observed for cell structure using a scanning electron microscope (SEM; MERLIN^®^ Compact, SEM-Zeiss, Munich, Germany).

### 2.7. Statistical Analysis

Experiment data were recorded in a spreadsheet program using Microsoft Excel. Descriptive statistics, including percentages, means, and standard deviations, were performed using the R programming language version 4.5.2. (https://www.r-project.org, accessed on 1 December 2025).

## 3. Results

### 3.1. Prevalence of M. pachydermatis and Antimicrobial Susceptibility of M. pachydermatis to Itraconazole

A total of 53 isolates (90.57%) were suspected to be *M. pachydermatis*. The prevalence of *M. pachydermatis* in dogs was 42% (95% CI = 28.19–56.79%). In addition, 45 isolates (84.91%; 95% CI = 72.41–93.25%) of suspected *M. pachydermatis* isolates were identified with a mean match score of 2.15 ± 0.17. The proportion of *M. pachydermatis* habituating in the ear side was 47.62% (95% CI = 25.71–70.22%) for bilateral ears and 53.38% (95% CI = 29.78–74.29%) for unilateral ears. The isolates were then tested for susceptibility to itraconazole. The MIC of itraconazole against 3-day-old *M. pachydermatis* ranged from 0.03–0.25 µg/mL. The mean MIC of itraconazole against the tested 3-day-*old M. pachydermatis* was 0.15 ± 0.09 µg/mL. Moreover, the MLC of itraconazole ranged from 0.03–0.25 µg/mL. The MIC_50_ and MLC_50_ of itraconazole were 0.125 µg/mL and 0.25 µg/mL, respectively, while the MIC_90_ and MLC_90_ were both 0.25 µg/mL. The mean MLC of itraconazole was 0.17 ± 0.10 µg/mL.

### 3.2. Phytochemicals of Crude Extract and Essential Oil of C. maxima Cultivar Tubtim-Siam Peel

**Figure 2:**
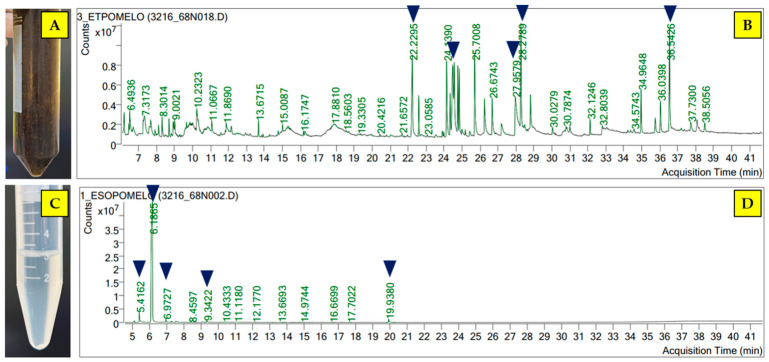
Gross appearance of the extracts and their GC-MS spectra of each extract using GC-MS of *C. maxima* cultivar Tubtim-Siam peel extract [(**A**) gross appearance of the crude extract; (**B**) GC-MS spectrum of the crude extract; (**C**) gross appearance of the essential oil; (**D**) GC-MS spectrum of the essential oil]. Dark blue arrows indicate the five most abundant phytochemicals in each extract.

Phytochemicals in the extract and the essential oil were identified using GC-MS. The ethanolic peel extract appeared a dark green, oily, viscous extract, while the essential oil presented a clear, viscous solution. GC-MS spectra of the crude extract and the essential oil assessed by GC-MS are presented in Figure 2. The crude extract primarily consisted of coumarins and fatty acids. Meranzin hydrate (6.46%) was the main compound identified in ethanolic peel extract. Furthermore, the main phytochemicals in the peel essential oil were terpenes, particularly D-limonene, which was the predominant component (79.75%). The proportion of identified phytochemicals in the essential oils and crude extracts is presented in Table 1 and Table 2, respectively.

### 3.3. Antimicrobial Activity of Crude Extract and Essential Oil of C. maxima Cultivar Tubtim-Siam Peel Against M. pachydermatis

**Figure 3:**
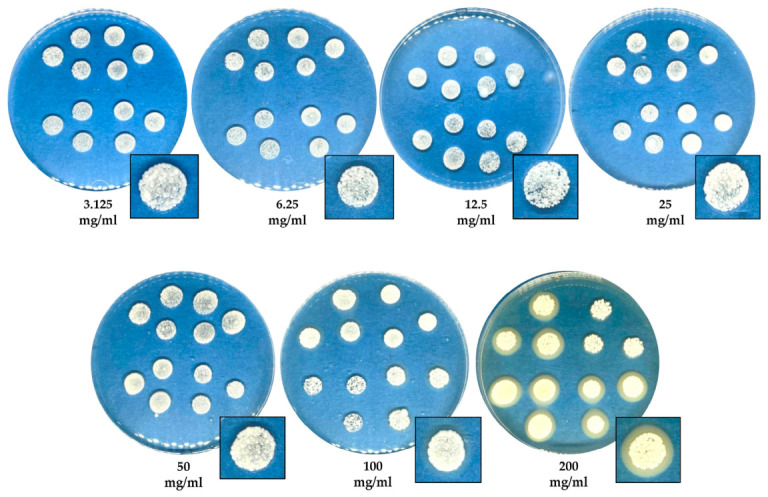
Agar plate dilution assay for *C. maxima* cultivar Tubtim-Siam peel crude extract (3.125–200 mg/mL) against *M. pachydermatis* growth (a blue background was used for the photograph; assay performed in triplicate).

**Figure 4:**
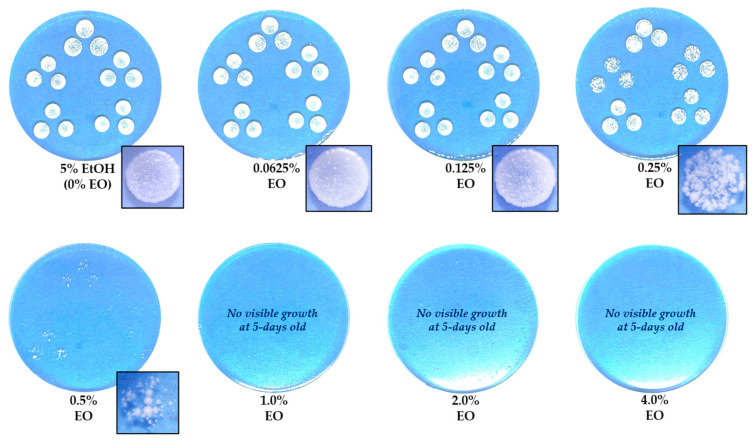
Agar plate dilution assay for *C. maxima* cultivar Tubtim-Siam peel essential oil (0–4% *v*/*v*) against *M. pachydermatis* growth (a blue background was used for the photograph; assay performed in triplicate).

The MIC and MLC values of the crude extract and essential oil of *C. maxima* cultivar Tubtim-Siam peel against *M. pachydermatis* were determined using an agar dilution assay. The results showed that the MIC for the crude extract exceeded 200 mg/mL; therefore, the MIC_50_ and MIC_90_ could not be determined. Figure 3 presents *M. pachydermatis* growth on agar containing crude pomelo peel extract in different concentrations. At higher crude concentrations (100–200 mg/mL), colonies altered the surrounding agar, presumably due to stress induced by high crude extract concentrations; however, even at 200 mg/mL, the crude extract exhibited no effect on growth inhibition. The MIC of essential oil ranged from 0.5–1.0% *v*/*v*. The concentration of 0.5% *v*/*v* essential oil inhibited the visible growth of *M. pachydermatis* after 3 days of incubation. In the 5-day experiment, a 1.0% *v*/*v* concentration of essential oil completely inhibited growth of *M. pachydermatis*, with no subsequent colony growth observed until the 7-day period of the experiment. In addition, the mean MIC of the essential oil was 0.89 ± 0.21% *v*/*v*. The MIC_50_ and MIC_90_ of essential oil were 1.0% *v*/*v*. The effect of essential oil on *M. pachydermatis* assessed using the agar plate dilution method is presented in Figure 4.

### 3.4. Effect of Prototypical Topical Essential Oil Solution on M. pachydermatis

**Figure 5:**
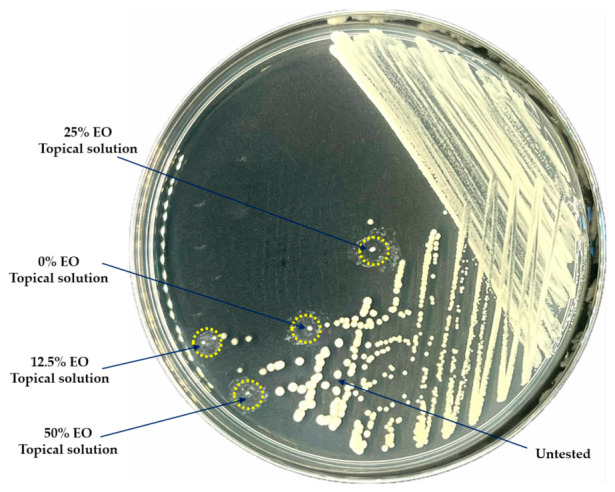
Colony characteristic of *M. pachydermatis* treated with prototypic topical solutions containing different concentrations of *C. maxima* cultivar Tubtim-Siam peel essential oil (yellow circles indicate treated colonies; each blue arrow points to a treated colony; assay performed in triplicate).

**Figure 6:**
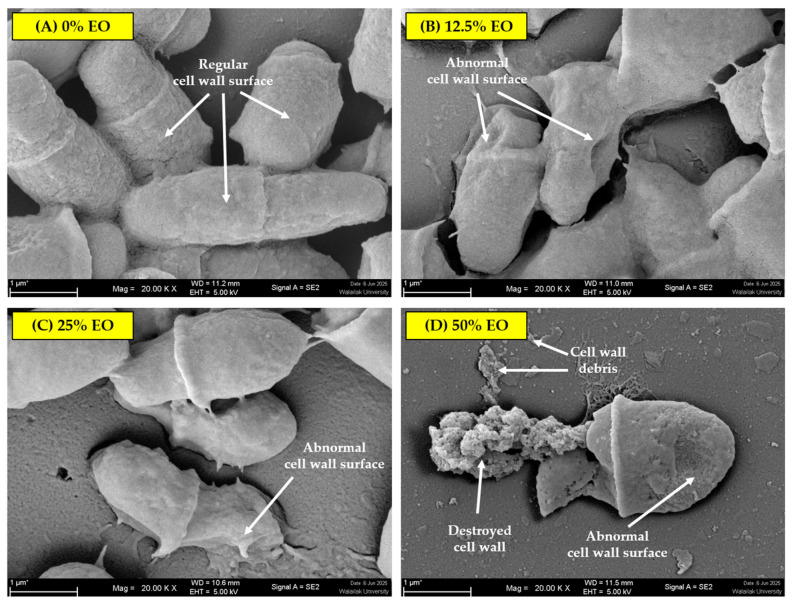
Cell surface morphology of *M. pachydermatis* treated with prototypic topical solutions containing different concentrations of *C. maxima* cultivar Tubtim-Siam peel essential oil, observed under a scanning electron microscope at 20,000× magnitudes [(**A**) 0% *v*/*v* essential oil; (**B**) 12.5% *v*/*v*; (**C**) 25% *v*/*v*; (**D**) 50% *v*/*v*; observations for each concentration were performed in duplicate].

Based on the antimicrobial activity of the essential oil, a prototypic topical solution was assessed against the pathogen. Then, *M. pachydermatis* was tested with different formulations of essential oil ear drops. The gross appearance of the treated colonies compared with untreated controls revealed that the colonies that were treated with higher essential oil concentrations showed disrupted colony structure at the end of the experiment. The treated colonies were smaller than the untreated colonies. Figure 5 shows a representative *M. pachydermatis* isolate. The morphology of the pathogen treated with the solution was observed for external cellular morphology under a scanning electron microscope. As shown in Figure 6, normal yeast cells with oval to rod-shaped morphology were detected in the control group. After treatment, the cell wall structure of *M. pachydermatis* differed from untreated control cells (0% *v*/*v* essential oil-containing prototypic topical solution). Moreover, the cell structure of *M. pachydermatis* was clearly disrupted by the 50% *v*/*v* essential oil treatment, compared with the control. In addition, shriveled cells were observed in the treatment groups. The appearance of *M. pachydermatis* cells in the different prototypic topical solution treatments is presented in Figure 6.

## 4. Discussion

Otitis externa in dogs is primarily caused by *M. pachydermatis* and is a widespread clinical concern [29]. The pathogen can cause zoonotic infections, particularly in immunocompromised humans [30]. Furthermore, infections caused by *M. pachydermatis* are difficult to treat due to its pathogenesis. Citrus fruits, including pomelo, are rich in bioactive compounds with antimicrobial activity [31]. This study found that different pomelo peel extraction methods produced distinct inhibitory effects against *M. pachydermatis*. In the ethanolic extract, coumarins were the predominant compounds identified. Meranzin hydrate was the predominant compound found in ethanolic extract. This phytochemical has also been identified in other plants, such as the flowers of *Magydaris tomentosa*, and exhibits antimicrobial activity at high concentrations, particularly against bacteria (*Enterococcus faecalis*, *Staphylococcus epidermidis*, *Staphylococcus aureus*, *Enterobacter aerogenes*, *Enterobacter cloacae*, *Escherichia coli*, *Klebsiella pneumoniae*, *Proteus vulgaris*, and *Salmonella* Typhi; MIC ≤ 128 µg/mL) [32]. Meranzin hydrate has also been reported in the peel of *C. maxima* [33]. Therefore, meranzin hydrate may be the primary phytochemical responsible for inducing stress in *M. pachydermatis* colonies in this study. However, the mechanism of action of meranzin hydrate remains unclear.

Pomelo peel essential oil exhibited inhibitory activity against *M. pachydermatis* at low effective concentrations. Limonene contains two active forms, including D-limonene and L-limonene [34], with D-limonene being the predominant biologically active form [35]. In this study, D-limonene was identified as the major phytochemical in the extracted oil. D-limonene is a monocyclic monoterpene commonly found in citrus fruits such as lemons, oranges, grapefruits [36], and pomelos. Approximately 80% of citrus peel essential oil consists of D-limonene [37], which is consistent with our findings. Furthermore, D-limonene was reported as an alternative phytochemical used for antimicrobial applications [38,39]. Previous studies have reported that D-limonene presented a broad antimicrobial spectrum, including antibacterial and antifungal activities [40], and can inhibit yeast growth in *Malassezia* and *Candida* [25,41,42]. Previous mechanistic studies in *Saccharomyces cerevisiae* suggest that D-limonene primarily affects ergosterol and β-1,3-glucan in the yeast cell wall [43,44]. In addition, D-limonene has been shown to interfere with ATP synthesis in *Candida* and *Malassezia* yeasts [45,46,47], and to inhibit the growth of *Candida tropicalis* by increasing membrane permeability, disrupting cell membrane integrity, and causing intracellular leakage [46]. Beyond antimicrobial activity, D-limonene has been reported to exhibit anti-inflammatory effects [48,49], which may enhance the therapeutic potential of essential oil–based topical formulations. However, these effects should be further validated using canine skin cell lines. In addition, D-limonene has been associated with skin-rejuvenating properties through oxidative stress reduction, modulation of inflammatory biomarkers, and angiogenesis [50,51].

A prototype topical solution containing *C. maxima* cultivar Tubtim-Siam peel essential oil exhibited inhibitory activity against *M. pachydermatis*, as confirmed by cell disruption observed using scanning electron microscopy. In this study, glycerol and propylene glycol were incorporated into the formulation to increase viscosity, thereby enhancing contact time between the bioactive compound and *M. pachydermatis*. However, this formulation represents a preliminary prototype requiring further optimization to improve its efficacy. Consistent with previous studies, D-limonene has been shown to affect cell membranes and cell walls, as demonstrated using scanning electron microscope and transmission electron microscopy [40]. Changes in cell wall thickness in both bacterial and fungal cells have been reported following D-limonene treatment [40]. The change of cell wall thickness of the cells of both bacteria and fungi was observed after D-limonene treatment [40]. Due to its hydrophobic and lipophilic properties, D-limonene alters membrane permeability, leading to leakage of intracellular contents and ultimately cell lysis [40,46,52,53]. D-limonene was the primary bioactive compound in the formulation and is classified as Generally Recognized as Safe (GRAS) by the U.S. Food and Drug Administration. It has also been widely used in cosmetic applications [40,54]. However, due to its high volatility [40], the inclusion of stabilizing agents to reduce evaporation is recommended. Various nanotechnologies, including nano-delivery systems, nano-emulsions, nano-gels, and nano-encapsulation, have been applied to improve stability and efficacy in agricultural and medical applications [40,55,56]. The stability of D-limonene remains a challenge for large-scale applications due to its hydrophobicity, susceptibility to oxidative degradation, and volatility under various conditions [57,58]. Therefore, D-limonene stability control needs to be performed, such as using polymer encapsulation technology [38]. This study experimented with the effect of essential oil and D-limonene in vivo on *M. pachydermatis* isolated from dogs; nevertheless, further studies on lab animals should be established for safety, clinical finding approval, and side effect determinations. The limitation of this study was the lack of in vivo testing on animal models and in vitro testing on skin cell lines. Due to several skin allergy reports of D-limonene [59,60], skin allergy testing for D-limonene-containing topical solutions should be further investigated. Furthermore, stability of the formulation should be carried out.

## 5. Conclusions

Essential oil of Tubtim-Siam pomelo peel (*C. maxima* cultivar Tubtim-Siam) contained antifungal activity against *M. pachydermatis*. The essential oil contained a high concentration of the bioactive phytochemical D-limonene that inhibits *M. pachydermatis* growth in vitro. The MIC of pomelo peel essential oil was 1% *v*/*v*, which inhibited growth over the 5-day experimental period. The anti-*M. pachydermatis* effect of the prototypical topical essential oil solution presented predominantly at 12.5% *v*/*v*. Furthermore, a 50% concentration of the essential oil in the formulation completely destroyed the yeast cells completely. The essential oil formulation affected the cell wall structure of the tested yeasts. Overall, the findings demonstrated that pomelo peel essential oil could be applied for veterinary applications, particularly as a topical agent. Further studies are needed to evaluate stability, mechanism of action, and suitability for industrial-scale applications.

## 6. Patents

This research received petty patent number 2503003645 regarding the “Formulation and preparation process of *Citrus maxima* cultivar Tubtim-Siam peel essential oil extract containing ear drop solutions.”

## Figures and Tables

**Table 1:** Phytochemical composition of the essential oil from *C. maxima* cultivar Tubtim-Siam peel.

Chemical Name of Essential Oil Extract	Chemical Formula	Relative Amount (%)	Classification
D-limonene	C_10_H_16_	79.75	monoterpenes
β-Pinene	C_10_H_16_	3.52	monoterpenes
trans-Linalool oxide	C_10_H_18_O_2_	1.36	oxolanes
Nootkatone	C_15_H_22_O	1.26	sesquiterpenes
L-α-Terpineol	C_10_H_18_O	0.82	monoterpenes
4-Carvomenthenol	C_10_H_18_O	0.27	terpenes
Neral	C_10_H_16_O	0.23	monoterpenes
Anhydrolinalool oxide	C_10_H_16_O	0.18	oxolanes
Citral	C_10_H_16_O	0.16	monoterpenes
α-Phellandrene	C_10_H_16_	0.14	monoterpenes
8-Hydroxyageraphorone	C_15_H_24_O_2_	0.10	sesquiterpenes
Others (<0.1% each)	-	12.20	-

**Table 2:** Phytochemical composition of the crude extracts from *C. maxima* cultivar Tubtim-Siam peel.

Chemical Name of Crude Ethanolic Extract	Chemical Formula	Relative Amount (%)	Classification
Meranzin hydrate	C_15_H_18_O_5_	6.46	coumarins
cis-Linoleic acid	C_18_H_32_O_2_	6.12	fatty acid
Yuehgesin C	C_17_H_22_O_5_	5.8	coumarins
Palmitic acid	C_16_H_32_O_2_	5.75	fatty acid
γ-Sitosterol	C_29_H_50_O	5.30	steroids
Isoauraptene	C_15_H_16_O_4_	4.21	coumarins
Arborescin	C_15_H_20_O_3_	4.18	sesquiterpenes
α-Linolenic acid	C_18_H_30_O_2_	3.97	fatty acid
Auraptenol	C_15_H_16_O_4_	2.55	coumarins
Linalool oxide	C_10_H_18_O_2_	2.53	monoterpenes
Ethyl α-linolenate	C_20_H_34_O_2_	2.42	fatty acid
Ethyl linoleate	C_20_H_36_O_2_	2.42	fatty acid
Hexadecanoic acid	C_19_H_38_O_4_	2.40	fatty acid
Cinnamic acid	C_12_H_14_O_4_	2.08	cinnamates
DL-α-tocopherol	C_29_H_50_O_2_	1.99	tocopherols
4-(1-amino-ethyl)-phenol	C_8_H_11_NO	1.91	phenols
1-(2,6-Dimethyl-4-propoxyphenyl)-2- methyl-1-propanone	C_15_H_22_O_2_	1.85	NA
Stigmasterol acetate	C_29_H_48_O	1.66	steroids
Ethyl hexadecanoate	C_18_H_36_O_2_	1.56	fatty acid
Phenol	C_6_H_6_O	1.48	phenols
Dihydroalatamide	C_16_H_17_NO_2_	1.43	benzamides
β-Methyl-D-glucoside	C_7_H_14_O_6_	1.31	glycoside
Ethyl 2,3-epoxybutyrate	C_6_H_10_O_3_	1.25	NA
Others (<1% each)	-	29.37	-

## Data Availability

The original contributions presented in this study are included in the article. Further inquiries can be directed to the corresponding author.
